# The impact of cognitive remediation on cerebral activity in schizophrenia: Systematic review of the literature

**DOI:** 10.1002/brb3.908

**Published:** 2018-02-07

**Authors:** Laura Bon, Nicolas Franck

**Affiliations:** ^1^ Centre Ressource de Réhabilitation psychosociale et de Remédiation Cognitive Centre Hospitalier Le Vinatier Lyon France

**Keywords:** cerebral activity, cognitive remediation, schizophrenia

## Abstract

**Context:**

cognitive remediation involves either intensive training of impaired functions or implementing strategies to compensate for these impairments. In cases of schizophrenia, both methods have demonstrated benefits in terms of behavior and cerebral activity. However, despite the major differences between these two approaches, their impact has not yet been compared.

**Method:**

We searched the PsychInfo, Pubmed, and ScienceDirect databases using the key words “cognitive remediation,” “schizophrenia,” “cerebral activity,” and “magnetic resonance imaging,” in order to select studies investigating the effects of cognitive remediation on patients with schizophrenia. The studies selected had to present their approach in detail and measure its impact in terms of both cerebral activity and cognitive function, both before and after therapy. We divided the studies into two groups, those using the strategy method and those using the training method.

**Results:**

Eight studies were included in the review, four for the strategy method (88 patients, 44 of whom underwent remediation) and 4 for the training method (87 patients, 43 of whom underwent remediation). The analysis of the results of this study indicates that the training method is capable of activating more the targeted brain areas than the strategy method. However, the latter appears to encourage more extensive activation of the cerebral networks.

**Discussion:**

The studies used for this review vary widely in terms of the imaging methods and protocol. However, differences were found between the two methods and lead us to suggest that further studies, with proper bias control, should be conducted to systematically compare the two approaches.

## 1 INTRODUCTION

Schizophrenia is strongly associated with cognitive impairments (Heinrichs & Zakzanis, 1998). Antipsychotic drugs can relieve the main symptoms of the disease but have no therapeutic effect on this cognitive impairment (Weisbrod, Kiefer, Marzinzik, & Spitzer, 2000) which explains the increasingly widespread use of cognitive remediation for schizophrenia. Cognitive remediation involves restoring cognitive function, through intensive, repetitive training, or compensating for the impairment by putting into place strategies to counterbalance the impairment with the aim of obtaining long‐term benefits and an improvement in day‐to‐day functioning (Kurtz, 2012). In cases where the aim was to restore function, the remediation focuses on a specific training of the impaired functions using computer or paper‐based exercises. This work can be performed at home or in an institution but must be regular and repetitive to restore the proper functioning of the affected cerebral areas. The training starts out at a low level of difficulty and increases gradually until the executive functions are reached (Subramaniam et al., 2012). In the case of strategy‐based remediation, the focus is on developing methods to compensate for the impairment experienced. Cognitive training is less focused on repetitions but is combined with strategy work and reflexive thought about everyday life (Wykes, Huddy, Cellard, McGurk, & Czobor, 2011). These two methods have both produced significant improvements in cognitive function, showing particular gains in terms of working memory, problem‐solving, and long‐term memory (Minzenberg & Carter, 2012). Furthermore, cognitive remediation has also been shown to have an impact on cerebral activity, with increased activity in the frontal and prefrontal regions, as well as in the anterior cingulate cortex (Isaac & Januel, 2016). Remediation compensates for impairments (use of regions of the brain other than those affected) but also partially restores activations which have previously been reduced (Ramsay & MacDonald, 2015). While the cerebral effects of cognitive remediation have already been widely reported in the literature, the underlying processes which bring about these benefits remain poorly understood, and the impacts of these two types of remediation have not yet been compared systematically.

This review aims to compare the results of published studies using these different methods in order to better understand the mechanisms involved, based on the hypothesis that the two types of remediation have different effects on cerebral activity. We conducted a systematic search for studies investigating the two remediation methods, categorized as either strategy implementation approaches or training of the impaired functions, based on a detailed analysis of the techniques used.

## 2 METHOD

We conducted a systematic search using the PRISMA criteria (Moher, Liberati, Tetzlaff, & Altman, 2009) in the PsychInfo, Pubmed, and ScienceDirect databases. The terms used for the search related to the type of remediation used “cognitive remediation,” the “schizophrenia” population and the measurement of cerebral activity with the key words “cerebral activity” and “magnetic resonance imaging.” Our inclusion criteria were as follows: we selected randomized studies investigating the effects of cognitive remediation in adults with schizophrenia; the effects of the remediation on cerebral activity and cognition had to have been assessed before and after remediation for the purposes of comparison; the remediation program had to be described in detail and focus on cognitive function and not solely on social cognition. Indeed, there are no simple training methods for impairment of social cognition, and it is therefore difficult to compare the two methods on this point.

Over the course of the study, a table was completed showing the main data from each article (remediation technique, population, participant groups, the control therapy, the task used to measure cerebral activity, detailed information on the cerebral effects, the target cognitive impact, and the actual cognitive impact). In order to analyze and compare the results, we divided the studies into two categories according to the two methods investigated as follows: strategy or training. The studies were assigned to the two categories based on the training method or strategy method after careful examination of the programs used. For the studies assigned to the strategy category, the therapist was involved in the rehabilitation process, providing the participants with strategies to use themselves to improve performance, which they can reproduce in their daily lives. For this type of therapy, participants received individual care management, tailored to their specific situation (Eack et al., 2009; Edwards, Barch, & Braver, 2010; Pu et al., 2014; Vianin et al., 2014). In some cases, this therapy was conducted in small groups. The group then served as a forum for sharing strategies. The aim was not necessarily to restore the cognitive functions but to help the participants to deal with them by focusing on the capacities they still have. In the training category, the therapist is less involved, leaving the patients to progress through a series of repetitive, targeted training exercises in small groups or at home (Bor et al., 2011; Haut, Lim, & MacDonald, 2010; Hooker et al., 2012; Subramaniam et al., 2014). The main goal was to work on the altered functions to improve the cognitive functioning. Therefore, strategies are not needed: the participants only work on their weaknesses. The level of therapist involvement, the repetitiveness, the involvement of strategies were used as a criterion for distinguishing between the two methods. We also split the analysis of the results according to the two methods;

## 3 RESULTS

**Figure 1:**
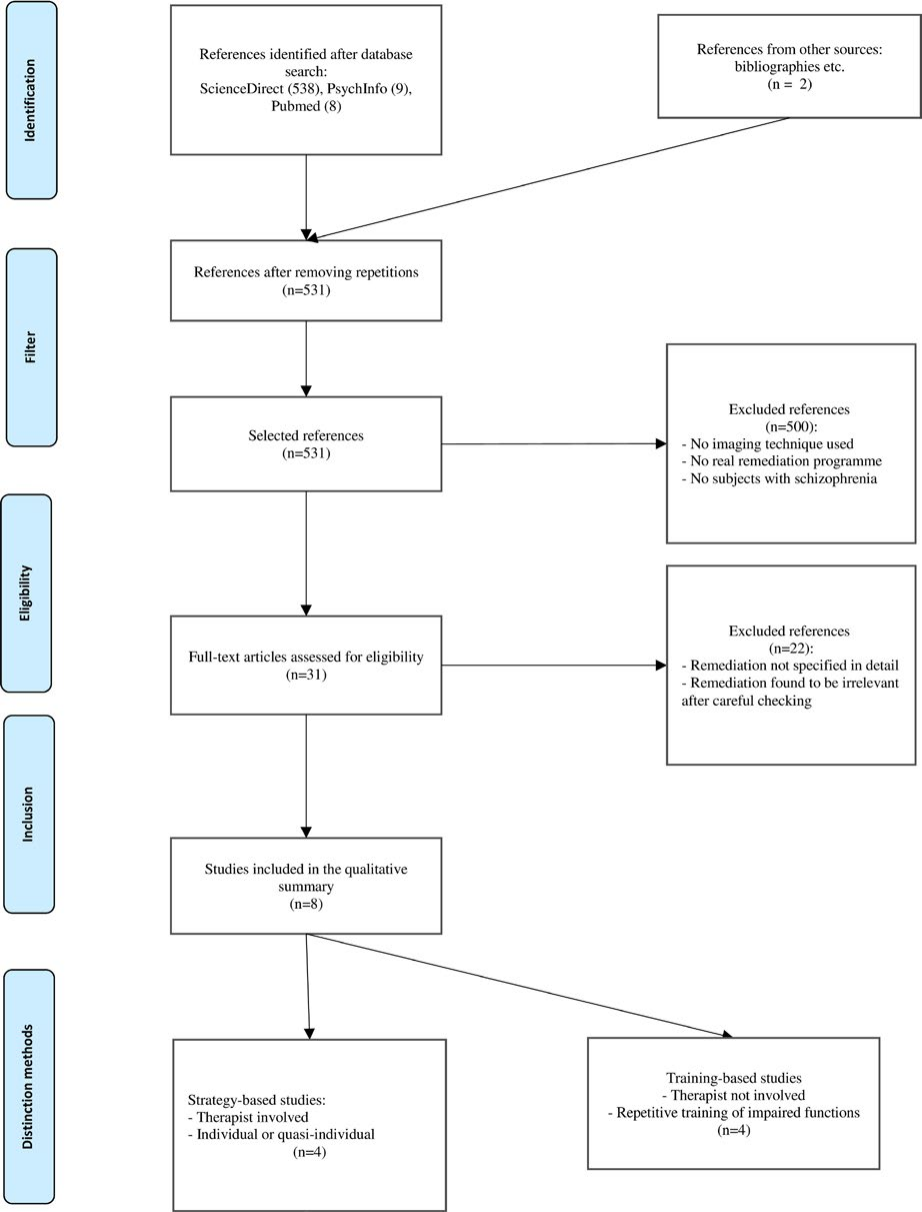
Prisma study selection flowchart

The search criteria are set out in the PRISMA study selection flowchart (Figure 1). The search found 555 articles. Five hundred and forty‐three studies were removed as they did not meet the eligibility criteria: use of imaging technique, use of a detailed cognitive remediation program, and patients with schizophrenia. Subsequently, any studies included twice over from the searches in different databases were removed, leaving a total of eight selected studies.

The patients included in these studies were stable and receiving treatment. Three studies demonstrated effects on connectivity, and a number of studies found correlations between increases in certain types of cerebral activity and improvements in cognitive function including attention, working memory, verbal memory, and cognitive control. These results are set out in detail in Tables 1 and 2.

**Table 1:** Summary of results, strategy method

Authors	Groups	MRI details	Duration/Frequency	Specific type of remediation	Cerebral impact of CR and functions of affected areas	Time to post CR evaluation	Neuropsychological improvements
Keshavan et al. (2016)	Sz CR or CR	Task: selective attention; ROI analysis	2 years: 60 hr software program + 40 hr social and cognitive therapy	Cognitive remediation software program (attention, memory, problem‐solving). + social and cognitive therapy groups	*↗ activity after CR*: dorsolateral prefrontal cortex → EF (planning, organization, flexibility, WM)* ↗ connectivity*: R dorsolateral PCF, posterior cingulate cortex associated with improvements in social cognition. *↘ connectivity*: R dorsolateral PFC, anterior cingulate cortex, and orbitofrontal cortex associated with improvements in cognitive function.	4 weeks	↗ verbal memory, EF,, and social cognition
Penadés et al. (2013)	Sz CR (*n *= 15) vs. Sz social skills training (*n *= 15) vs. healthy control subjects (*n *= 15)	Task: n‐back (WM)	16 weeks: 2–3 one‐hour sessions per week	CR with implementation of flexibility and WM strategies.	*↗activity Sz CR compared to the control*: Bilateral inferior and middle frontal gyrus Bilateral anterior cingulate gyrus → attention, motivation, verbal initiation bilateral middle temporal gyrus → auditory processing of language sounds Bilateral cingulate gyrus → attention, motivation, verbal initiation Bilateral precuneus → episodic memory, visuospatial processing, conscience Bilateral inferior occipital gyrus → visual attention Bilateral lingual gyrus *Other*: normalization of cerebral activity in people with schizophrenia after remediation → increased interhemisphere connectivity between the left and right prefrontal cortex ↘ in activity in regions which were previously hyperactive in comparison with healthy subjects.	2 days	↗ EF (categorisztion, planning), verbal, and non‐verbal memory
Pu et al. (2014)	CR vs. TAU	Task: 2‐back; analysis focused on the frontotemporal regions	24 weeks: 2 hr CR/week + 1 hr group/week	Software program (attention, memory, executive functions) + group discussion on applying strategies	*↗ in activity measured before and after CR*: Bilateral dorsolateral PFC (BA 9, 46) → EF (planning, organization of thought, flexibility) WM L ventrolateral PFC (BA45, Broca's area) → Broca language production R frontopolar (BA 10) → EF (planning, multitasking), WM. *Correlations: ↗ in activity* ventrolateral PFC and improvement in WM	No deadline	↗ verbal memory, processing speed, executive functions
Vianin et al. (2014)	Sz CR (*n *= 8) vs. Sz TAU (*n *= 8)	Task: verbal fluency	14 weeks: 2 hr CR/week + 14 hr at home	Computer‐based and paper program supported by a therapist (selective attention, visuospatial attention, visuospatial memory, reasoning, verbal memory, WM).	*↗ activity*: R inferior parietal lobule—162v → language ventral stream, resolution of spatial tasks Bilateral precentral gyrus—69v (AB 44) → voluntary movement, motor response L inferior frontal gyrus—29v (AB 45) → language production, verbal WM, EF (executive control of language) L middle occipital gyrus—15v → visual attention L midcingulate cortex—12v → attention, motivation, verbal response initiation L superior parietal lobule—10v → attention, dorsal stream visual system	1–2 months	↗ in inhibition and reasoning ability

CR: cognitive remediation; Sz: patients with schizophrenia; TAU: treatment as usual; WM: working memory, STM: short‐term memory; LTM: long‐term memory; v: number of voxels; EF: executive functions; PFC: prefrontal cortex; vmPFC: ventromedial prefrontal cortex; mPFC: medial prefrontal cortex; L: left; R: right; BA: Brodmann Area; ROI: Region Of Interest.

**Table 2:** Summary of results, training method

Authors	Groups	MRI details	Duration/ Frequency	Specific type of remediation	Cerebral impact of CR and functions of affected areas	Time to post CR evaluation	Neuropsychological improvements
Bor et al. (2011)	Sz CR (*n *= 8) vs. control (*n *= 9) + healthy group (*n *= 15)	Task: 2‐back (WM); exclusion of motor zones	6 weeks: 4 hr/week	Training software program (attention, memory, WM, executive functions).	*↗ activity*: L middle frontal gyrus—1006v → problem‐solving, initiation L inferior frontal gyrus—1006v (BA 44/45, Broca area) → initiation language L cingulate gyrus—413v (BA 24) → attention, motivation, verbal response initiation L inferior parietal lobule—346v → ventral stream (language), resolution of spatial tasks* Correlation*: ↗ Broca area and attention capacity	3 weeks	↗ attention and reasoning
Haut et al. (2010)	Sz CR (*n *= 9) vs. Sz TAU (*n *= 9) vs. healthy (*n *= 9)	Task: n‐back word, n‐back photo (WM); ROI analysis	4–6 weeks: 4–6 hr/week	Remediation software program (attention, memory, executive functions) and training using n‐back (WM). CR in groups of 4. Limited feedback from investigator.	*↗ activity before and after CR—task WM word*: L and R frontopolar cortex—273v and 134v (BA 10) → STM, EF (planning, multitasking) L dorsal PFC—1034v and L dorsolateral—57v (BA 6, 8, 9, and 46) → EF (planning, organization, flexibility), STM Anterior cingulate gyrus—498v (BA 24/32) → attention, motivation, verbal initiation *↗ activity before and after CR—task STM photo*: L and R frontopolar cortex—535v and 585v (BA 10) → STM, EF (planning, multitasking) R dorsal PFC—623v and L dorsal—593v (BA 6, 9) → EF (planning, organization, flexibility), STM Anterior cingulate gyrus—458v (BA 24/32) → Attention, motivation, verbal initiation —insular cortex—457v (BA13) →pleasure system, somatosensory area	1–2 weeks	↗ n‐back performance (WM)
Hooker et al. (2012)	Sz CR + cognitive and social therapy (*n *= 11) vs. Sz video games (*n *= 11)	Task: emotion recognition analysis based on hypothesis of involved regions	10 weeks: 5 hr software program/week + approximately 1 hr of social and cognitive therapy / week	Remediation software program for WM and processing of auditory and verbal information (speed, attention, effectiveness) in laboratory or at home. + emotion recognition training	*↗activity*: R postcentral gyrus—35v (BA 3) → somatosensory function, reception of temporal info R supplementary motor area—23v (BA 6) → planning complex tasks Bilateral precentral gyrus—24v and 18v (BA 3) → voluntary movement, motor response R superior temporal gyrus—17v (BA 48) → auditory processing and comprehension L angular gyrus—39v (BA 39) → complex language processing R superior frontal gyrus—15v (BA 9) → EF (planning, WM, action regulation)	NA	↗ in facial emotion recognition capacity
Subramaniam et al. (2014)	CR vs. video games vs. healthy TAU	Task: n‐back (WM)	16 weeks: 7 hr/week	CR software program visual processing, auditory–verbal, WM, and social cognition	*↗ in activity at 16 weeks*: L middle frontal gyrus → EF (problem‐solving, verbal fluency, risk‐taking) L inferior frontal gyrus → language production, verbal WM, EF (executive control of language) *↗connectivity at 16 weeks*: R middle frontal gyrus, L middle frontal gyrus, and L inferior frontal gyrus. *Correlation*: ↗ R middle frontal gyrus ↗ WM performance	No deadline	↗ LTM, EF

CR: cognitive remediation; Sz: patients with schizophrenia; TAU: treatment as usual; WM: working memory, STM: short‐term memory; LTM: long‐term memory; v: number of voxels; EF: executive functions; PFC: prefrontal cortex; vmPFC: ventromedial prefrontal cortex; mPFC: medial prefrontal cortex; L: left; R: right; BA: Brodmann Area; ROI: Region Of Interest.

### 3.1 Strategy method

**Figure 2:**
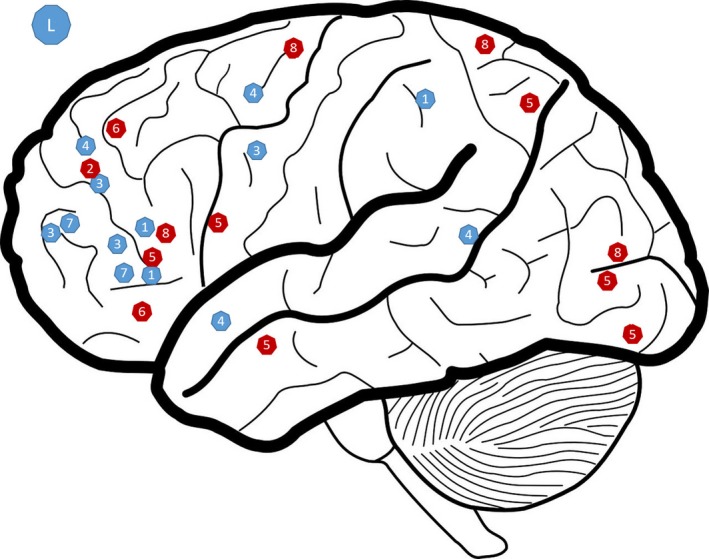
Representation of zone showing significantly high levels of activation after CR in the left hemisphere (R). This figure is a qualitative representation. In red: strategy; in blue: training; 1: Bor et al. (2011); 2: Keshavan, Eack, Prasad, Haller, and Cho (2016); 3: Haut et al. (2010); 4: Hooker et al. (2012); 5: Penadés et al. (2013); 6: Pu et al. (2014); 7: Subramaniam et al. (2014); 8: Vianin et al. (2014)

**Figure 3:**
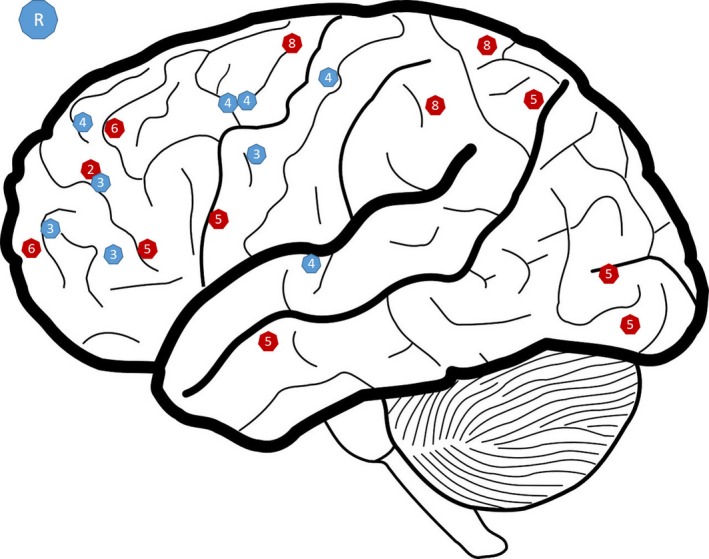
Representation of zone showing significantly high levels of activation after CR in the right hemisphere (R). This figure is a qualitative representation. In red: strategy; in blue: training; 1: Bor et al., 2011; 2 : Keshavan et al. (2016); 3: Haut et al. (2010); 4: Hooker et al. (2012); 5: Penadés et al. (2013); 6: Pu et al. (2014); 7: Subramaniam et al. (2014); 8: Vianin et al. (2014)

The four studies focusing on strategy implementation included a total of 88 patients, 44 receiving cognitive remediation and 44 control therapies. In each study, a therapist was present throughout the sessions during which patients worked through exercises on a computer using a purpose‐designed software program or on paper. Discussion of the strategies also sometimes took place in groups to encourage participants to develop and share their strategies. Generally, the aim was to help them develop methods that they could use in their everyday life. The duration of the programs varied from 14 to 45 weeks with between 2 and 4 hr of therapy per week. The increased cerebral activity observed after remediation was mainly concentrated in the frontal regions in the middle and inferior frontal gyrus (Vianin et al., 2014), the precentral gyrus (Vianin et al., 2014), the dorsolateral prefrontal cortex (Keshavan et al., 2016; Pu et al., 2014), the midcingulate cortex (Penadés et al., 2016; Vianin et al., 2014), the ventromedial prefrontal cortex (Pu et al., 2014), and the frontopolar cortex (Pu et al., 2014; Vianin et al., 2014). Increases in activity were also found in the parietal and occipital lobes, the inferior and superior parietal lobule (Vianin et al., 2014), precuneus (Penadés et al., 2016), the middle and inferior occipital gyrus (Penadés et al., 2016; Vianin et al., 2014), and the lingual gyrus (Penadés et al., 2016). Increased cerebral activity was also found in the temporal lobe in the middle temporal gyrus (Penadés et al., 2016). These results are set out in detail in Table 1. Two of the studies presented searched regions of interest in the anterior cingulate cortex and midcingulate cortex, as well as in the superior and medial prefrontal cortex (Keshavan et al., 2017b; Pu et al., 2014), and the other analyses took into account the whole brain. The different activations observed are shown in Figures 2 and 3.

### 3.2 Training method

The four studies investigating training of impaired functions included 74 healthy individuals and 87 patients, 43 of whom undertook training and 44 were given a control therapy. The exercises were completed in the laboratory or at home, and therapists were sometimes present but were not supposed to intervene. The programs were intense and repetitive. They varied in duration from 4 to 16 weeks, with 4–7 hr of remediation per week. Only one study was conducted by region of interest (Haut et al., 2010), and the others excluded certain motor regions or covered all cerebral activity. Generally speaking, in the training studies, the increases in cerebral activity were observed in the prefrontal regions: inferior, superior, and middle frontal gyrus (Bor et al., 2011; Hooker et al., 2012; Subramaniam et al., 2014), anterior cingulate gyrus (Bor et al., 2011; Haut et al., 2010), frontopolar cortex (Bor et al., 2011; Haut et al., 2010), dorsolateral prefrontal cortex (Haut et al., 2010; Pu et al., 2014), precentral, and postcentral gyrus (Subramaniam et al., 2014). Some temporal regions were also affected, including the superior temporal gyrus and the angular gyrus (Hooker et al., 2012). The inferior parietal lobule and the insular cortex also showed increased activations through remediation (Bor et al., 2011; Haut et al., 2010).

The different activations observed are shown in Figures 2 and 3. This figure presents a visual overview of the activations found in the different studies included in this review. However, it is not necessarily representative as not all studies considered the brain as a whole.

### 3.3 Risk of bias

The risk of bias in the studies included in this review is presented in Table 3. We used the Cochrane criteria to evaluate these risks. All the studies included were randomized, and some were double blinded.

**Table 3:** Description of risk of bias

	Randomised selection of subjects	Blinded (participants)	Blinded (investigator not informed of effects sought)	Results reported in full
Bor et al. (2011)	Yes	No	Yes	Yes
Haut et al. (2010)	Yes	No	NA	Yes
Hooker et al. (2012)	Yes	Yes	Yes	Yes
Keshavan et al. (2016)	Yes	NA	NA	Yes
Penadés et al. (2013)	Yes	No	Yes	Yes
Pu et al. (2014)	Yes, but groups not randomised	NA	NA	Yes
Subramaniam et al. (2014)	Yes	Yes	Yes	No
Vianin et al. (2014)	Yes	No	Yes	NA

NA, not available.

## 4 DISCUSSION

This literature review compared the cerebral impact of cognitive remediation techniques based on the implementation of strategies, against those based on the repetitive training of impaired functions. The aim was not only to review the existing literature on the topic but also to underline the different mechanisms raised by these two methods. Indeed, some studies are already reviewing the cerebral impact of cognitive remediation. Yet, no comparisons were made between the methods themselves. All the remediation techniques presented in this review had a significant impact on the participants' cerebral activity. Remediation increases the activation of the cerebral regions which support executive functions, regardless of the method used. The two cognitive remediation methods do, however, have different effects in terms of the intensity of the increase in activation and the locations of these activations. The training method is capable of activating more the targeted brain areas than strategy‐based techniques. The training‐based remediation sessions focus on specific cognitive functions and involve the related cerebral regions, as subjects are asked to concentrate on a specific task. Therefore, this result is coherent with the method itself: a specific training leads to specific increasing of cerebral activations. The number of voxels mobilized should be interpreted with caution as the different studies measured activity at different time intervals postremediation, using different methods and devices. However, the difference observed remains noteworthy.

As regards the locations of the effected regions, only studies using the strategy method saw increases in activity in areas such as the precuneus, involved in episodic memory (Cavanna & Trimble, 2006; Yokoyama et al., 2010). However, this function was also targeted by some of the training method studies (Haut et al., 2010; Subramaniam et al., 2014). This is an interesting finding because behavioral improvements in relation to memory are also observed for this type of remediation, which therefore indicates the involvement of other cerebral regions. Furthermore, the strategy method activates more of the zones responsible for executive functions. It would appear that implementing strategies activates a broader network, involving cerebral areas not related to the target functions, the lingual gyrus, for example, which governs visual attention (Penadés et al., 2013) and the superior parietal lobule which is involved in the dorsal stream of the visual system (Vianin et al., 2014). This hypothesis is backed up by studies analyzing connectivity, as the strategy method results in better connectivity across wider networks than training techniques which remain concentrated around the prefrontal cortex. This result is coherent with the strategy‐based training which is less focused and involves more cerebral regions than the training method.

It is difficult to compare data on improvements in cognitive function as several studies did not measure cognitive function after remediation. However, the studies which did include these measurements show improved performance in verbal memory, short‐term memory, long‐term memory, and executive functions, regardless of the type of remediation used.

The main limitations of this review result from the fact that the studies analyzed were not designed to be compared. We have contextualized the regions in which the activity was modified by remediation. Some studies were conducted as regions of interest analyses which make it difficult to conclude on the extent of the impact of remediation. It is possible that the training studies produced other activations, in addition to those recorded. Furthermore, comparing the intensity of activation is hindered by the fact that some studies did not present their results in voxels. The fact that the measurements were not taken at the same time after remediation may also have affected the results. Finally, we mainly based our categorization of the studies on the nonintervention of the investigator. However, it is possible that even using the training method the investigator interacted with participants, recreating the conditions specific to more strategic remediation approaches. The heterogeneity of the results obtained makes it difficult to draw comparisons between the two remediation methods. In order to overcome these difficulties, it would be interesting to set up a randomized study to compare the two methods with strict control over the criteria for each method in order to avoid investigator bias and with the same method for measuring cerebral activity, in order to compare the regions activated. The evaluations of cerebral activity should also be performed at the same time interval postremediation.

Previous studies have already reviewed the effects of cognitive remediation on cerebral activity and linked them with behavioral improvements. The present review is the first to investigate the different existing remediation methods in order to compare their results on brain activations. Strategy‐based remediation methods are very different from the training methods, and these two types of remediation probably have different effects on cerebral activity. This literature review showed a greater increase in activity obtained using the training method, but with a wider activation network for the strategy method. Understanding the cerebral mechanisms underlying the behavioral improvements obtained would allow us to optimize patient management in cognitive remediation (Wykes et al., 2011).

## ACKNOWLEDGMENT

The authors thank Mrs Kim Barrett for editing the manuscript and Mr Julien Plasse for his help concerning the figures.

## CONFLICT OF INTEREST

None declared.
